# T220. THE GLUTAMINASE INHIBITOR EBSELEN PREVENTS AMPHETAMINE SENSITIZATION IN MICE

**DOI:** 10.1093/schbul/sby016.496

**Published:** 2018-04-01

**Authors:** Lior Heffetz-Giterman, Sharon S Lander, Reut Cohen, Amit Gross, Inna Gaisler-Salomon

**Affiliations:** University of Haifa

## Abstract

**Background:**

Dysregulated glutamatergic neurotransmission has been strongly implicated in the pathology of schizophrenia (SZ). Glutaminase 1 (GLS1) plays a critical role in the recycling of glutamate. GLS1 deficient mice were previously shown to display an attenuated response to the acute and chronic effects of the dopamine-releasing psychotomimetic drug amphetamine and have a pro-cognitive profile. A recent large-scale drug screening study identified ebselen as a potent CNS-available GLS1 inhibitor. Here, we asked whether ebselen (10 mg/kg) would attenuate sensitization to amphetamine (4 mg/kg) and induce pro-cognitive behavior.

**Methods:**

Sensitization to amphetamine (4mg/kg) was tested in the open field. Mice received either saline, amphetamine or amphetamine+ebselen (10mg/kg) i.p. on 4 consecutive days. Seven days later, mice were challenged with amphetamine, amphetamine+ebselen or saline. We further assessed the effect of ebselen administration on Gls1 mRNA in the hippocampus, prefrontal cortex and striatum, and on dopamine receptor expression in the striatum. Finally, we measured social preference and recognition in genetically modified GLS1 deficient mice and in ebselen (10mg/kg)-treated wild-type mice.

**Results:**

We found decreased sensitization to amphetamine in mice that received pre-treatment with ebselen. Gene expression studies revealed reduced Gls1 expression in hippocampus, and altered expression of dopamine markers in the striatum of ebselen-treated mice. Finally, ebselen-treated mice show enhanced social recognition, similarly to GLS1 deficient mice.

**Discussion:**

Similarly to genetically modified GLS1 deficient mice, ebselen-treated mice demnstrate resilience to the sensitizing effects of the pro-psychotic drug amphetamine and a pro-cognitive phenotype. These findings provide evidence for the potential of GLS1 inhibition in addressing some of the central clinical features of SZ and related pathology.

